# Drop Test Kinematics Using Varied Impact Surfaces and Head/Neck Configurations for Rugby Headgear Testing

**DOI:** 10.1007/s10439-022-03045-5

**Published:** 2022-08-24

**Authors:** Danyon Stitt, Natalia Kabaliuk, Keith Alexander, Nick Draper

**Affiliations:** 1 Department of Mechanical Engineering, University of Canterbury, Private Bag 4800, Christchurch, 8140 New Zealand; 2 Faculty of Health, University of Canterbury, Christchurch, New Zealand; 3 Sport Health and Rehabilitation Research Centre (SHARRC), University of Canterbury, Christchurch, New Zealand

**Keywords:** Concussion, Impact method, Rugby, Rotational accelerations, Linear accelerations, Impact recreation

## Abstract

World Rugby employs a specific drop test method to evaluate headgear performance, but almost all researchers use a different variation of this method. The aim of this study was, therefore, to quantify the differences between variations of the drop testing method using a Hybrid III headform and neck in the following impact setups: (1) headform only, with a flat steel impact surface, approximating the World Rugby method, (2 and 3) headform with and without a neck, respectively, onto a flat MEP pad impact surface, and (4) headform and neck, dropped onto an angled MEP pad impact surface. Each variation was subject to drop heights of 75–600 mm across three orientations (forehead, side, and rear boss). Comparisons were limited to the linear and rotational acceleration and rotational velocity for simplicity. Substantial differences in kinematic profile shape manifested between all drop test variations. Peak accelerations varied highly between variations, but the peak rotational velocities did not. Drop test variation also significantly changed the ratios of the peak kinematics to each other. This information can be compared to kinematic data from field head impacts and could inform more realistic impact testing methods for assessing headgear.

**Supplementary Information:**

The online version contains supplementary material available at 10.1007/s10439-022-03045-5.

## Introduction

During a game of rugby players are exposed to a large number of head impacts, imparting high linear and rotational accelerations to the head.^28–32^ Studies by King *et al.* found that players receive an average of 14–52 significant [above 10 g peak linear acceleration (PLA)] impacts to the head per game.^28,31,32^ It is therefore not surprising, that one of the most common injuries in rugby is a mild traumatic brain injury (mTBI), commonly labeled as concussion.^16,21,39^ Several studies report mTBI rates during rugby gameplay of up to 46 injuries per 1000 athlete exposures,^13,38,43,51,52^ however, mTBI under-reporting rates have been estimated to be as high as 50–90%.^15^ The problem deepens, however, as one study of ice hockey players found significant structural changes in the white matter diffusivity of the corpus callosum between pre and post-season brain scans compared with a control group of non-contact sports players.^40^ The magnitude of change was associated with poorer verbal learning and memory. It should be noted that none of the ice hockey players reported any concussive injuries over that season. A similar study of American football players found players diagnosed with an mTBI, along with 50% of those with no diagnosed mTBI showed significant alterations in brain function during fMRI scans.^7,55^

Kinematic measures of head motion during impacts, such as PLA, peak rotational acceleration (PRA), and peak rotational velocity (PRV), are commonly reported to quantify brain injury risk for several reasons. Most notably; the relative ease of measurement, as accelerometers are affordable and widely available, and that the kinematics offer an indication of the brain’s inertial response.^49^ It has long been known that rotational kinematics alone elicit much larger structural damage to the brains of primates,^6,8,9,17,23^ whilst several studies have found that linear kinematics alone did not correlate well with diagnosed brain injury on field.^20,22,26^ Outside a laboratory setting, however, head impacts are unlikely to be purely linear or rotational. How the combined linear and rotational kinematics relate to brain injury in humans has not been made clear, leading to the development of various injury metrics based on measurable linear and rotational kinematics. The most well-known of which are: the head injury criterion (HIC), rotational injury criterion (RIC), head impact power (HIP), brain injury criterion (BrIC), and risk curves developed by Rowson and colleagues.^27,44,49,54,56^ Unfortunately, despite their commonplace in brain injury research, most single variable metrics (PLA, *etc*.) and injury criterion alone do not provide region-specific or temporal characteristics of brain mechanical response believed to induce strain-based brain tissue injury.^25^ To date, the best predictors of human brain injury likelihood within the literature are the peak regional brain, and axonal, strains,^22,50^ based on FE modeling of singular head impacts, followed by the peak rotational kinematics (PRV and PRA).^26,34^

Most contact sports mandate headgear to protect players from skull and brain injuries. Rugby is not one of those sports. Headgear use has not been made mandatory by World Rugby, and until recently, the development of protective headgear has been limited by the strict laws imposed by World Rugby.^58^ The requirements for headgear approval have been relaxed in the more recent Law 4 trial assessment,^59^ allowing new types of headgear to be developed showing promising results for brain injury reduction in the laboratory.^10,14,53^. Specifically, the limit on material density (previously limited to ≤ 45 kg/m^3^) and prohibition of sandwich construction were lifted. Despite this change, the testing methods used to evaluate impact attenuation remain largely unchanged. The World Rugby standard requires headgear, fitted to a steel headform conforming to EN960,^11^ to be dropped onto a steel impact surface with an impact energy of 13.8 J. Impact attenuation is limited such that impacts must produce a PLA above 200 g. Such a limit has been introduced as players are encouraged to protect themselves rather than use equipment to materially provide injury protection.

Despite the compounding body of literature stating PLA does not correlate well with brain strain, it is the only parameter evaluated in either standard. In the newer trial standard, the drop heights for impact attenuation testing have been changed to 0.15, 0.3, 0.45, 0.6, and 0.9 m, with the attenuation limit also removed. Despite removing this limit, no clear criteria have been detailed for impact attenuation performance of headgear in this trial standard.

Several investigations detailing the acceleration reduction offered by rugby headgear have been published,^10,12,14,33,42,53^ but only three are relevant to this study. The first, by Ganly *et al.*,^14^ assessed their (at the time) newly developed Npro headgear using the methods set in the World Rugby law 4 testing process. This study quantified the PLA reduction of the headgear during these tests, however, extended this analysis to the rotational domain using a separate test setup. The methods used to generate these rotational accelerations were not described in depth in the paper or in the paper referenced for the test setup.^48^ In 2021, we studied the ability of rugby headgear to reduce the peak accelerations using a drop test method based on the NOCSAE standard^10,45^ Impacts were carried out on a wire-guided drop test rig using a Hybrid III (HIII) 50th percentile male headform. The headform was instrumented with a single triaxial accelerometer at the COM of the headform and only measured linear acceleration. The final study details our more in-depth analysis of rugby headgear, extending the investigation into the rotational domain.^53^ This study furthered the equipment and methods employed by Draper *et al.* to include impacts that were non-centric (direction of impact force does not travel through the COM) onto angled surfaces inducing large amounts of shear and rotational behavior. Headform instrumentation was also upgraded to include a “Nine Accelerometer Package” (NAP) for calculating rotational accelerations^47^ along with the inclusion of a HIII neck.

There are no standards available for assessing soft-shelled headgear in the rotational domain. Even research groups investigating hard-shelled headgear (with more widely used standards) create individual drop test methods to represent more realistic impact conditions in the lab^1–5,41^ All of which used a combination of moving impact surfaces or angled impact surfaces of varying compositions. Only three studies fully report differences in drop test variations. One investigated the effect on the PLAs of impact mass and speed in a drop test.^19^ The second quantified the effect on linear and rotational kinematics of headform shape and neck use between a HIII and NOCSAE headform.^3^ The last of these considered the effect on the linear and rotational accelerations of impact surface compliance, impact location, and impact velocity.^46^ These studies, however, failed to consider several important details. Bland *et al.* reported differences in the temporal profile of the kinematics but used only a single impact velocity. Additionally, the authors only tested the headforms with bicycle helmets attached. No study investigated the effects of impact surface angle or quantified the differences in rotational velocity.

This gap in knowledge needs filling as many researchers, companies, and governing bodies (such as World Rugby) continually use different variations of the drop test to evaluate headgear performance. Therefore, this study aimed to quantify differences in the kinematic behavior of four drop test variations representing the World Rugby standards, NOCSAE standards, and work by the previously mentioned research groups.

## Materials and Methods

### Experimental Procedure

All impacts were carried out on a twin wire-guided drop test rig using a HIII headform instrumented with a nine accelerometer package (NAP). Four variations of drop test method were used for comparison (Table 1). The first aimed to represent, as close as possible, the drop test method implemented by World Rugby.^58^ This used the HIII headform, with no neck, and a steel impact surface (130 mm diameter, 40 mm height). Drops onto the steel impact surface were limited to 300 mm maximum height to reduce damage to the headform and components. The second impact setup recreated a study by Draper *et al.* and used a HIII headform with no neck and a 1-inch MEP pad impact surface.^10^ The third and fourth drop test setups recreated our more recent headgear study.^53^ Both were carried out with a HIII headform and HIII neck onto a 1-inch MEP pad impact surface angled at 0° and subsequently at 45° relative to the ground. The 30° impact surface was excluded from this analysis under the assumption that differences in kinematic headform response between a flat (0°) and 30° impact surface would be the same (albeit different magnitude) as those between a 0° and 45° impact surface. All impacts onto the MEP pad were limited to 600 mm drop height to reduce damage to the headform and internal components. Further detail on our previous methods is found in the referenced articles. Table 1 summarises the different drop test conditions used with the associated drop height, impact velocity, and impact energy. It should be noted that the mass used for calculating the impact energy includes the headform, drop carriage, and neck (when used).

Headgear was excluded from this study to focus the results purely on the bare headform response. Impacts onto the 45° impact surface were assumed to create motions that were only allowable by a flexible neck. The occipital condyle (OC) joint on the HIII head does not permit such movement, therefore, 45° impacts without the neck were excluded from the study. Impacts with and without the neck were assumed to produce similar kinematic differences independent of the impact surface. Additionally, drops onto steel are inherently harsh on testing components. To reduce the risk of damage we minimized the number of these impacts, thus excluding impacts with the neck onto both flat and 45° steel impact surfaces. Excluding these impact setups was additionally favored to minimise cluttering of the results. Impacts onto the 0° impact surface from 75 mm drop height were the last impacts carried out. Unfortunately, damage to an adaptor plate (from carrying out steel impacts) halted data collection for impacts with the neck onto the 0° MEP pad from this height. Fortunately, enough data had already been collected to generate valid results.

**Table 1:** Drop test conditions.

Impact surface	Drop heights (mm)		Impact velocity (m/s)		Impact energy (J)	
	Neck	No neck	Neck	No neck	Neck	No neck
0° MEP pad	–	75	–	1.2	–	4.2
	150	150	1.7	1.7	9.8	8.4
	300	300	2.4	2.4	19.6	16.8
	450	450	3.0	3.0	30.6	26.3
	600	600	3.4	3.4	39.3	33.8
45° MEP pad	150	–	1.7	–	9.8	–
	300	–	2.4	–	19.6	–
	450	–	3.0	–	30.6	–
	600	–	3.4	–	39.3	–
0° Steel	–	75	–	1.2	–	4.2
	–	150	–	1.7	–	8.4
	–	225	–	2.1	–	12.9
	–	300	–	2.4	–	16.8

Dashes indicate drop test conditions that were excluded from the study

**Figure 1:**
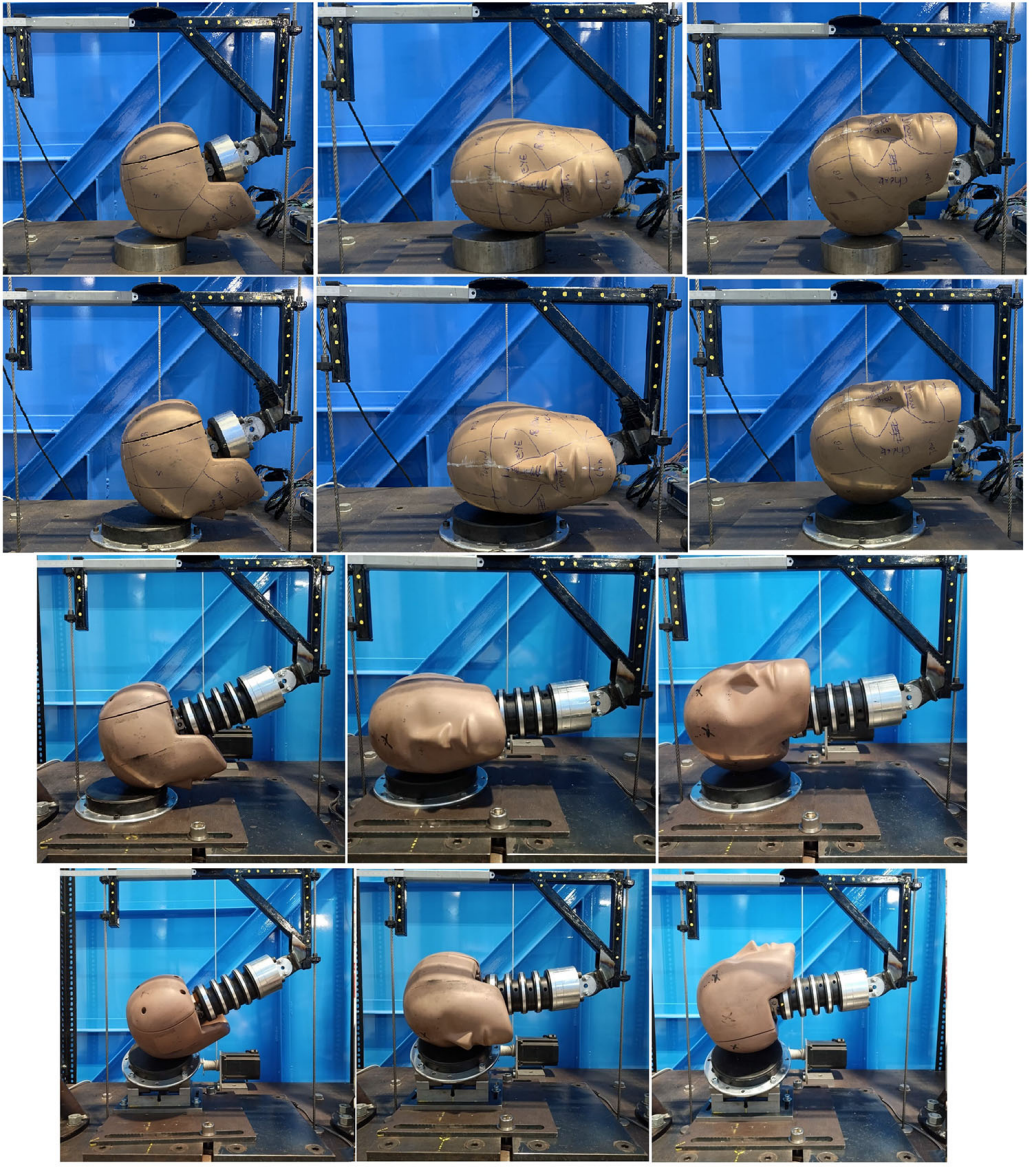
Impact test setup for each drop test variation. The top row shows the steel impact surface with no neck attached. The second and third rows show the flat (0°) MEP pad impact surface with no neck, and including the neck, respectively. The bottom row shows the (45°) impact surface with the neck attached. From left to right, all rows show the forehead, side, and rear boss impact locations.

Three impact orientations were used for this study; the forehead, side, and rear boss. Every impact scenario, shown in Table 1, was subjected to each of the three orientations. These orientations (shown in Fig. 1) were chosen as they comprised the set of orientations that could be created on both flat and angled impact surfaces, and covered the highest and lowest kinematic response for given impact energy. All impacts were carried out twice with 60–70 s between successive drops.

### Data Acquisition and Post Processing

The HIII headform was instrumented with four triaxial accelerometers (Analog Devices ADXL377, 20,000 Hz, range: ± 200 g, sensitivity: 6.5 mV/g) creating a NAP with three redundant sensing axes, to measure linear and rotational accelerations.^47^ Data from the accelerometers was recorded by a LabVIEW system for 4 s, with the impact event recorded in the middle. This long-duration data recording ensured data was captured from the release of the headform from the electromagnet until long (around 1500 ms) after impact. Recording in such a way allowed extra validation and verification steps during post-processing.

Accelerometer data was post-processed using Python 3.8. To validate each impact, accelerometer data (in m/s^2^) was integrated, using NumPy’s cumtrapz function, giving linear velocities (m/s). The peak pre-impact velocities of each accelerometer were compared to the theoretical velocity (from Eq. (1)), where *v* is the impact velocity, *g* is gravity (9.81 m/s^2^), and *h* is the drop height in m. Impacts were discarded and recreated if any peak linear velocity differed by more than 5% compared to the other accelerometers, or the theoretical value. The temporal location of peak linear velocity also indicated the instant of impact. All accelerometer data before this time was set to zero, reducing the cumulative errors associated with integration. All accelerometer data exceeding 200 ms post-impact was deemed insignificant to the study and was also set to zero. Additionally, at longer post-impact durations, the effect of the drop carriage and other external features become significant. As these effects were outside the scope of this study, they were ignored.

1 $$\begin{aligned} v = \sqrt{2 g h} \end{aligned}$$

Comparison of the shapes of the kinematic traces was carried out by first trimming the data for each impact to start 20 ms pre-impact and end 200 ms post-impact. A 220 ms window was used based on two studies of the dynamic characteristics of brain strain during impact.^24, 37^ Both studies found that long-duration (above 100 ms) data recording was needed to simulate an appropriate brain strain response which included the peak strain values. Resultant kinematic traces were subsequently normalized based on the peak value, following which the mean kinematic profile shape was found. This was found by taking the mean and standard deviation of each data point across the range of heights for each impact variation. Peak values (PLA, PRA, and PRV) were found from the resultant linear acceleration, rotational acceleration, and rotational velocity respectively. Peak kinematic results are presented as the mean peak value across the three orientations tested for each impact variation.

### Statistical Analysis

Both linear and nonlinear regressions were used to find relations between the peak values and the impact velocity and impact energy. All nonlinear regressions took the form of Eq. (2)

2 $$\begin{aligned} y = ax^b \end{aligned}$$

where *a* and *b* were determined using Python’s curve-fitting package (SciPy.optimize, curve_fit) using the least-squares method. The function in Eq. (2) was chosen as it is constantly increasing for positive values of *a* and *b*. A constant increase was assumed to make sense physically, outside the range encompassed by the data in this study. Additionally, the curve was forced through the origin for all cases. The goodness of fit was quantified using $R^2$ defined by Kvalseth for nonlinear regression.^35^ Regression analysis was carried out on each of the three orientations for each impact scenario. The regressions are reported as the mean (with standard deviation) of the parameters *a* and *b* taken from averaging across the three impact orientations. Significant differences between regression lines were not found.

## Results

### Effect of Impact Surface Compliance

**Figure 2:**
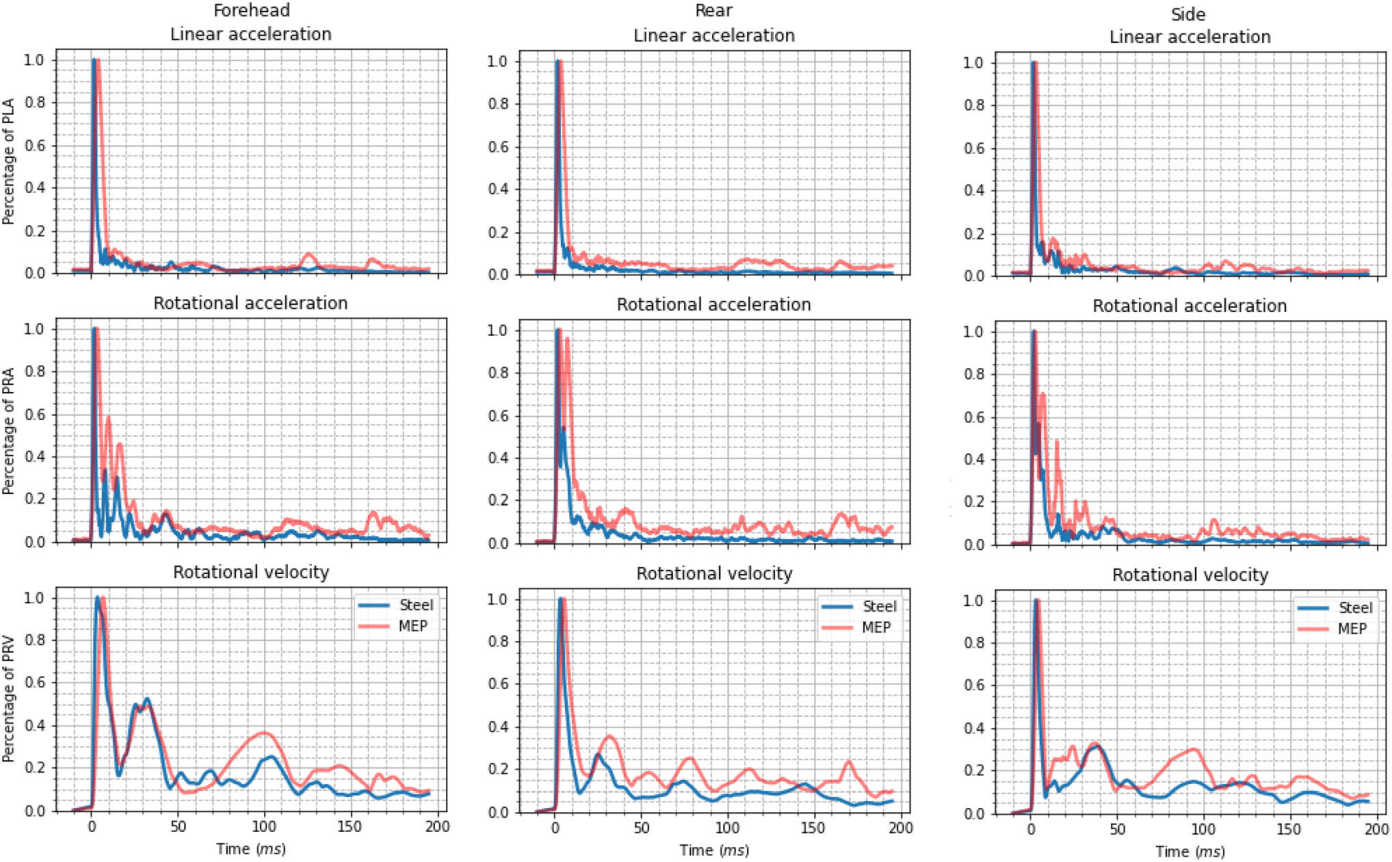
Standardized resultant kinematic profiles resulting from impacts onto a steel impact surface (blue, following World Rugby), and a 1-inch MEP pad impact surface (red). From the top row to the bottom row, the plots show the linear acceleration, rotational acceleration, and rotational velocity.

Figure 2 shows the mean kinematic profiles generated from impacts onto steel and MEP impact surfaces. Both impact conditions excluded the neck and were onto a flat (0°) impact surface. Unsurprisingly, drop tests onto steel resulted in larger PLAs than those onto the MEP pad ($56\pm 5$–$188 \pm 7$ g for steel vs $31 \pm 2$–$82 \pm 9$ g for the MEP pad). Durations of the linear acceleration peak also varied between the two impact methods, with the MEP pad creating longer linear acceleration peaks ($6.1 \pm 1$ ms for steel and $11.5 \pm 0.7$ ms for the MEP pad).

Impacts onto the MEP pad created rotational kinematics with heightened “secondary peaks” compared to the steel impact surface. For clarity, secondary peaks describe an additional peak in the acceleration or velocity profiles immediately following the primary peak. Due to these secondary peaks, rotational acceleration peak duration was excluded from the analysis. Fortunately, visual differences alone provided significant information about differences between impact conditions. For the rear boss orientation impacting an MEP pad, the rotational acceleration displayed a secondary peak nearly the same magnitude as the main peak. The duration of rotational kinematics did not differ greatly between the steel and MEP impact surfaces. Despite the MEP pad impacts reaching PRV later than the steel counterpart, there is little difference between the rotational velocity profiles.

### Effect of Including the Neck

**Figure 3:**
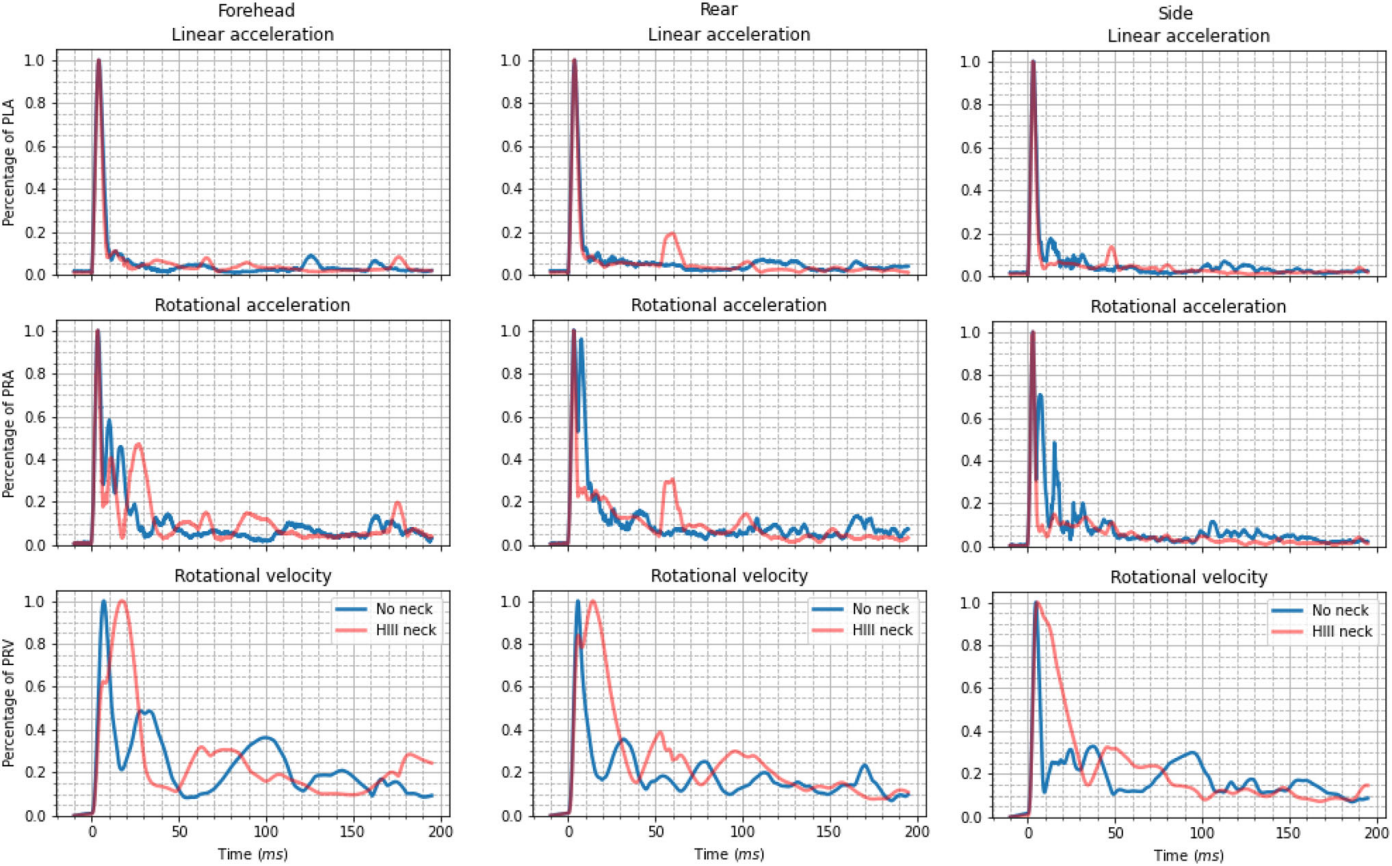
Standardized resultant kinematic profiles resulting from impacts with the neck (blue), and without the neck (red), onto a $0^\circ$ MEP pad. From the top row to the bottom, the figure shows the linear acceleration, rotational acceleration, and rotational velocity.

Figure 3 shows differences in kinematic profile between impacts with and without the neck, onto a $0^\circ$ MEP impact surface. Impacts without the neck reached PLA after those with the neck ($3.8 \pm 0.3$ ms vs. $3.6 \pm 0.4$ ms). Correspondingly, impacts without the neck displayed longer duration linear acceleration peaks ($11.5 \pm 0.7$ ms) than those including the neck ($9.8 \pm 0.2$ ms). Whilst both impact conditions reached PRA at $3.4 \pm 0.2$ ms, the acceleration profiles differed significantly. Impacts without the neck produced large secondary rotational acceleration peaks in the rear and side orientations. This was not seen during drop tests with the neck, however, secondary impacts were more common with the neck.

Small peaks in linear acceleration that occurred after the primary peak were labeled “secondary impacts”. These manifested when the headform experienced a significant “rebound” following the initial impact. When such behavior occurred, the headform returned to the impact surface creating the secondary impacts seen in Fig. 3. Secondary impacts created perturbations in all three kinematic profiles but were most clear in the linear acceleration profiles. Secondary impacts are not to be confused with secondary acceleration peaks, which occurred without a secondary impact.

Forehead impacts created more similar rotational acceleration profiles between the two impact conditions. Rotational velocity profiles differed significantly between the two impact setups. Impacts without the neck reached PRV earlier ($5.8 \pm 0.9$ ms vs. $12.4 \pm 5$ ms) and displayed shorter duration rotational velocity peaks ($15.5 \pm 4.2$ ms vs. $40.5 \pm 5.7$ ms) than impacts with the neck. Large differences were observed in post-peak behavior, with neck impacts showing much larger rotational velocities following the initial peak. This, however, was most likely due to the earlier incidence of secondary impacts when including the neck.

### Effect of Impact Surface Angle

**Figure 4:**
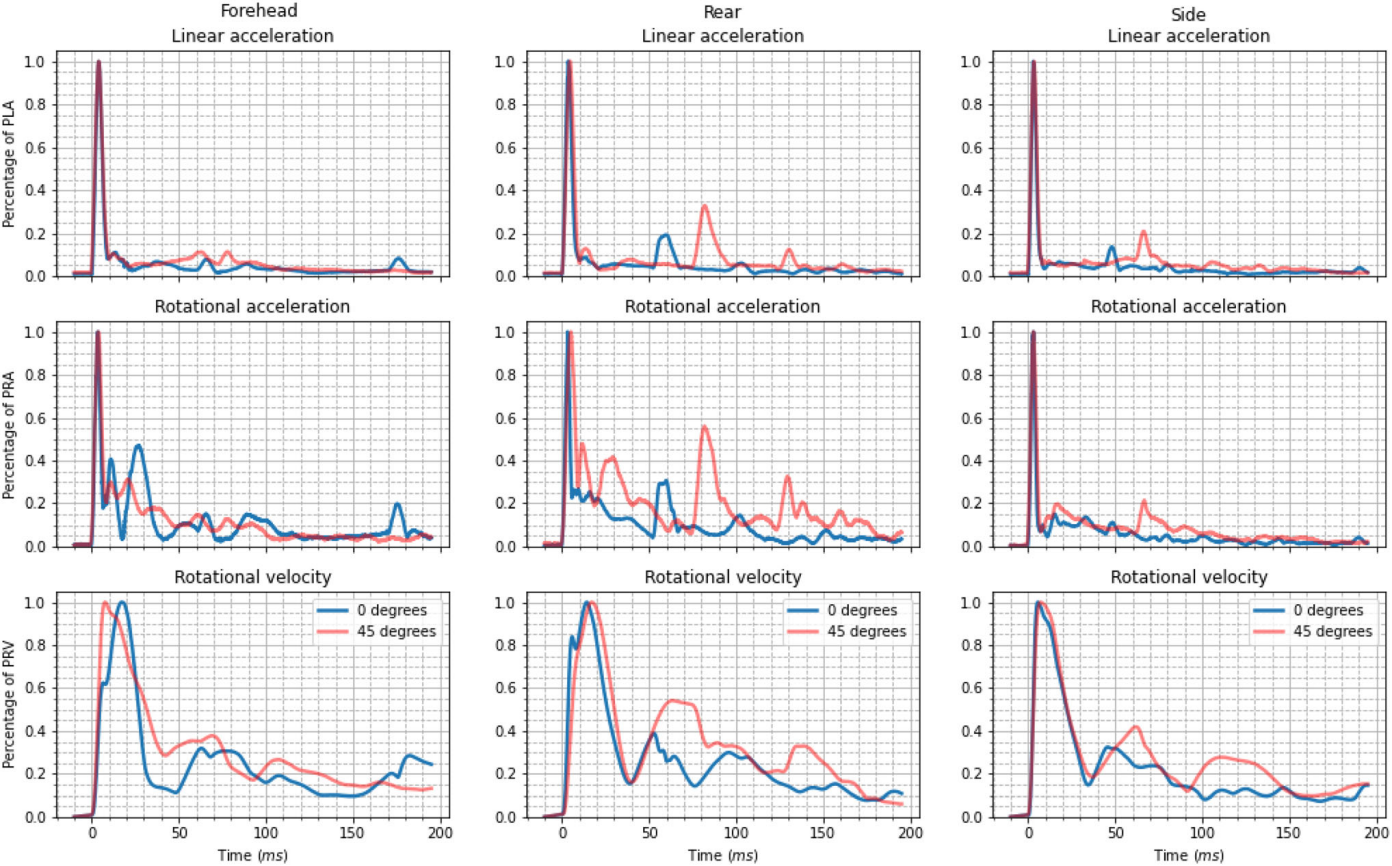
Standardized resultant kinematic profiles from impacts onto a flat $0^\circ$ (blue) and angled $45^\circ$ (red) impact surface. Both impact variations include the neck. From top row to bottom, the figure shows linear acceleration, rotational acceleration, and rotational velocity.

Minimal differences were seen in the linear acceleration profiles between impacts onto the $0^\circ$ and $45^\circ$ MEP pad (Fig. 4). The duration of the linear acceleration peak did not differ between the two impact conditions($9.6 \pm 0.8$ ms for $0^\circ$ and $9.8 \pm 0.2$ ms for $45^\circ$ impacts), nor did the time to reach PLA ($3.6 \pm 0.4$ ms for $0^\circ$ and $4.1 \pm 0.4$ ms for $45^\circ$ impacts). Large secondary impacts were recorded for both 0 and $45^\circ$ impacts on the forehead and side orientations. Impacts onto the $0^\circ$ impact surface displayed this secondary impact much earlier than impacts onto the $45^\circ$ surface.

Post-peak rotational acceleration profiles differed significantly between the 0 and $45^\circ$ impact surface for the forehead and rear orientation. Forehead impacts onto a $45^\circ$ surface heavily reduced the magnitude of secondary rotational acceleration peaks compared to the $0^\circ$ MEP impacts. Conversely, $45^\circ$ impacts in the rear orientation produced larger secondary peaks than those onto the $0^\circ$ surface in the rotational acceleration profile. The side orientation remained largely unaffected by the change in impact surface angle.

Despite the differences in rotational accelerations, the rotational velocity profiles were strikingly similar. The times to reach PRV were similar, with flat impacts taking slightly longer than angled impacts ($10.8 \pm 4.6$ ms for $45^\circ$ and $12.4 \pm 5.0$ ms for $0^\circ$ impacts). Duration of the rotational velocity peak was nearly identical ($39.9 \pm 2.3$ ms for $45^\circ$ and $40.6 \pm 5.7$ ms for $0^\circ$ impacts). Post-peak rotational velocity behavior remained relatively similar between the two impact conditions, with relatively high velocities observed until the 200 ms time.

### How are Peak Values Affected by Method Variations?

**Figure 5:**
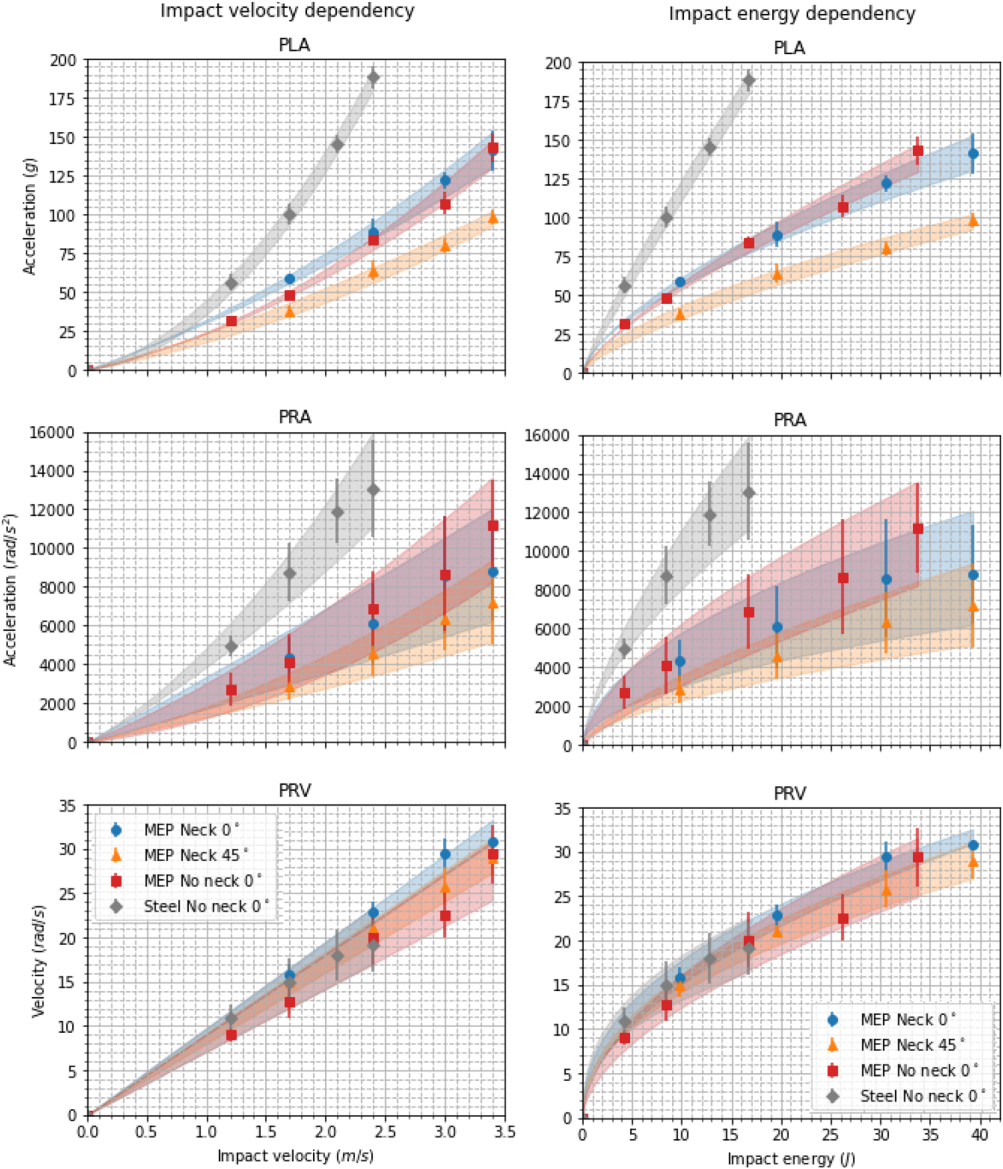
Composite peak resultant values of linear acceleration (top), rotational acceleration (middle), and rotational velocity (bottom) for the different impact velocities (left) and impact energies (right) used. Shaded areas show the mean ± SD of the regression functions across the impact orientations.

Figure 5 shows the composite mean PLA, PRA, and PRV, with their dependence on the impact velocity and impact energy. Composite means were calculated by averaging the peak values across the three impact orientations for each drop test condition.^10,53^ Although impact velocity and impact energy are related by $E = \frac{1}{2}mv^2$, both are reported here for ease of comparison with different impact testing setups. Unsurprisingly, all peak kinematics increased as the impact velocity and energy increased. PLA accounted for the largest variation in the data between different drop test conditions. For every impact condition, PLAs and PRAs increased nonlinearly, and the rate at which they increased for each impact condition varied highly. Steel impacts showed the highest PLAs and PRAs for given impact energies, whereas impacts with the neck onto a $45^\circ$ surface showed the lowest PLAs and PRAs for given impact energies. Figs. 7 and 8 in Supplementary materials show this held true across all orientations. Minimal differences were observed between peak kinematics of impacts with and without the neck onto the $0^\circ$ MEP surface. PRVs increased linearly with impact velocity and did not vary significantly between drop test conditions. When considered for impact energy, the same behavior was observed.

Table 2 displays the mean (SD) parameters *a*, *b* optimized for peak kinematic value for each impact condition. All peak kinematics were strongly correlated with their respective regression functions ($R^2 = 0.97{-}0.99$). Most surprisingly, the PRVs were mostly unaffected by variation in the drop test condition. Additionally, PRV was strongly linearly correlated with impact velocity, and strongly, although nonlinearly correlated to, impact energy. Steel impacts showed the highest PLAs for a given impact velocity and energy with a mean regression function of $PLA = 38.84vel^{1.78}$ $R^2 = 0.99$, whereas the $45^\circ$ impacts showed the lowest PLAs with regression of $PLA = 18.83vel^{1.34}$ $R^2 = 0.99.$

A similar trend was seen with PRAs where steel impacts display consistently higher values, increasing at a greater rate than all other impact conditions. Impacts onto the 45° MEP pad displayed the lowest PRAs, which increased at the lowest rate compared to all other impact conditions. Interestingly, PLAs resulting from impacts with the neck onto the 0 and 45° MEP pad generated nearly identical values of power parameter *b* (1.34 (0.07) and 1.29 (0.09) respectively). Values of regression parameter *a*, however, differed between the two [29.07 (1.30) and 18.18 (2.00) for 0 and 45° MEP impacts]. This similarity did not extend past the linear domain.

Interestingly, regression parameter *b* for all peak kinematics with respect to impact energy was half that of regression parameter *b* for peak kinematics with respect to impact velocity. This was observed for all drop test conditions and all three kinematic variables. This makes sense as energy is proportional to the square of velocity.

### Kinematics Dependence

**Figure 6:**
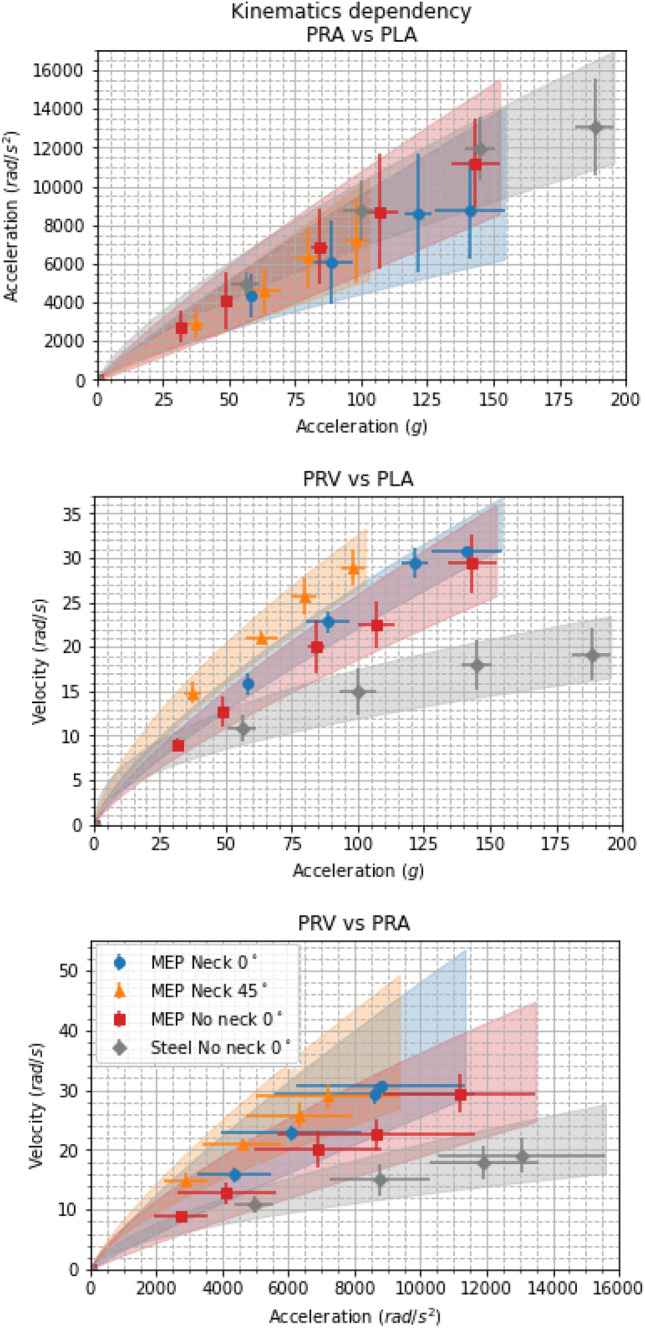
Figure shows how the composite peak resultant kinematics relate to each other. From top to bottom, the peak rotational acceleration with respect to the peak linear acceleration (PLA), the peak rotational velocity as a function of PLA, and the peak rotational velocity with respect to the peak rotational acceleration.

Figure 6 shows how the peak kinematic variables relate to each other for each impact condition. PRAs and PLAs were strongly correlated ($R^2 = 0.98{-}0.99$) and did not differ greatly across the drop test conditions. Thus implying the PRA is primarily a function of the PLA. This is reasonable as the linear kinematics were used to calculate rotational kinematics. The PRVs were also strongly non-linearly correlated with PLA values ($R^2 \ge 0.99$) and PRA values ($R^2 \ge 0.99$). Figure 9 in Supplementary materials shows how the peak kinematic variables were related for each impact orientation.

Neck $45^\circ$ impacts consistently displayed higher PRVs for the same PLA values compared with all other drop test setups, whilst steel impacts consistently created the lowest PRVs. Both drop test setups with a 0° MEP pad impact surface displayed similar PRVs for given PLAs. Steel impacts showed the lowest PRV values for PRA values, and neck 45° impacts showed the highest. It should be noted that the difference between all MEP pad impacts is relatively small. Table 2 shows the mean (SD) curve fitting parameters for each individual impact condition used to generate Figs. 5 and 6.

**Table 2:** Regression parameters and $R^2$ values for each impact condition. All functions take the form $y=ax^b.$

*x*	Impact velocity (*m*/*s*)			Impact energy (*J*)			PLA (*g*)		PRA $(rad/s^2)$
*y*	PLA (*g*)	PRA ($rad/s^2$)	PRV (rad/s)	PLA	PRA	PRV	PRA	PRV	PRV
Neck 45°									
*a*	18.83 (2.00)	1452.13 (223.00)	8.57 (0.57)	8.33 (1.21)	655.30 (54.50)	5.00 (0.38)	103.57 (13.24)	1.31 (0.13)	0.05 (0.0002)
*b*	1.34 (0.07)	1.29 (0.12)	1*	0.67 (0.04)	0.64 (0.06)	0.48 (0.02)	0.92 (0.07)	0.68 (0.02)	0.73 (0.03)
$R^2$	0.99 (< 0.01)	0.99 (< 0.01)	0.99 (< 0.01)	0.99 (< 0.01)	0.99 (< 0.01)	0.98 (0.01)	0.99 (0.01)	0.99 (0.01)	0.99 (< 0.01)
Neck flat									
*a*	29.07 (1.30)	2490.30 (755.46)	9.35 (0.30)	13.27 (1.31)	1303.42 (383.88)	5.50 (1.19)	171.50 (83.29)	0.95 (0.56)	0.013 (0.0097)
*b*	1.29 (0.09)	1.05 (0.02)	1*	0.64 (0.05)	0.53 (0.009)	0.48 (0.06)	0.82 (0.05)	0.75 (0.13)	0.90 (0.12)
$R^2$	0.99 (< 0.01)	0.98 (0.01)	0.99 (0.01)	0.99 (< 0.01)	0.98 (0.01)	0.99 (< 0.01)	0.99 (0.01)	0.99 (< 0.01)	0.99 (< 0.01)
No neck									
*a*	21.80 (1.37)	1969.86 (947.50)	8.07 (0.96)	9.75 (0.96)	954.89 (573.43)	3.82 (0.60)	139.69 (134.16)	0.65 (0.18)	0.02 (0.01)
*b*	1.51 (0.08)	1.46 (0.22)	1*	0.75 (0.04)	0.73 (0.11)	0.57 (0.01)	0.98 (0.17)	0.77 (0.04)	0.79 (0.07)
$R^2$	0.99 (< 0.01)	0.98 (< 0.01)	0.98 (< 0.01)	0.99 (< 0.01)	0.98 (< 0.01)	0.99 (< 0.01)	0.99 (< 0.01)	0.99 (< 0.01)	0.99 (0.01)
Steel									
*a*	38.84 (4.25)	4196.00 (778.30)	8.36 (1.30)	15.02 (2.34)	2107.01 (558.13)	6.21 (1.23)	324.43 (157.58)	1.87 (0.70)	0.08 (0.02)
*b*	1.78 (0.08)	1.33 (0.26)	1*	0.89 (0.04)	0.66 (0.13)	0.41 (0.05)	0.74 (0.14)	0.46 (0.06)	0.59 (0.02)
$R^2$	0.99 (< 0.01)	0.98 (0.01)	0.98 (< 0.01)	0.99 (< 0.01)	0.98 (0.01)	0.99 (< 0.01)	0.98 (0.02)	0.99 (< 0.01)	0.99 (< 0.01)

Data is displayed as the mean (SD) regression parameter and $R^2$ for the three impact locations of each impact condition. The regression functions describe the areas shown in Figs. 5 and 6

* indicates where a linear regression was used with the form $y = ax$

## Discussion

### Kinematic Profiles

All drop test setups resulted in different linear and rotational kinematics. How these differences manifest varied across the drop test conditions used. Impacts onto steel created shorter duration linear accelerations than identical drop tests onto the more compliant MEP pad. Stiffness of the impact surface had the largest effect on the linear acceleration duration with more compliant surfaces resulting in lower PLA, and longer duration accelerations. This makes sense as a more compliant impact surface accommodates a larger deformation during impact, resulting in a reduced PLA, but increased impact duration. Although different impact conditions varied the linear acceleration response, only minimal differences existed between impacts onto the MEP pad.

Rotational acceleration profiles varied more significantly by changing the drop test condition than the linear counterpart. An obvious difference was the presence of secondary peaks in the rotational acceleration profiles. Secondary peaks occurred using steel and MEP pad impact surfaces, however, including the neck reduced the magnitude of secondary peaks. One thing to note is that secondary acceleration peaks are opposite in direction to the primary peak. Resultant values are, by definition, always positive, therefore, secondary peaks appear in the same direction as the primary peak. With this understood, their explanation becomes clearer. When excluding the neck, secondary peaks occurred when the headform reached the maximum range of motion (ROM) permissible by the OC joint. Once this limit was reached, a large deceleration of the headforms rotational motion about the OC occurred, giving rise to the secondary acceleration peak.

Secondary peaks were less frequent when the neck was included, directly resulting from an increased permissible ROM. During impacts, the headform could rotate past the maximum ROM of the OC join. The extra ROM provided by the flexible neck damped the decelerations seen at the maximum ROM. The result was much lower magnitude secondary peaks than those seen during impact without the neck.

Whilst steel and MEP pad impact surfaces both produced secondary peaks, impacts onto the more compliant MEP pad resulted in much larger secondary peaks than those onto steel. This was likely a result of the MEP pad allowing larger elastic deformations during impact than the steel. The headform skin and impact surface both experience deformation during impact. As no permanent deformation of either occurred during impact, an elastic collision could be assumed. The combined elastic deformation of the headform skin and MEP pad during impact were larger than those of the headform skin and steel. The result was a higher velocity rebound off the MEP pad than the steel impact surface, allowing larger rotational velocities and, correspondingly, larger decelerations at the maximum ROM of the OC join.

These secondary peaks should not be confused with secondary impacts. Secondary impacts occurred when the headform rebounded from the initial impact and returned to the impact surface within the observed time window. Secondary impacts were characterized by distinct, and separate, linear acceleration peaks post primary acceleration peak. Additionally, these were clearly disjointed from the main acceleration peak. This behavior was most common when the neck was included in the drop test condition, and when impacting the rear of the head.

For clarity, almost all impacts resulted in a secondary impact. Altering the impact condition changed the time between the primary and secondary impacts. The neck allowed a larger ROM post-impact but also resulted in less rebound from the impact surface. Due to the decreased rebound height, the headform could achieve a secondary impact much earlier than impacts without the neck.

One thing to note is that impacts onto a flat surface created secondary impacts that were much closer (temporally) to the main peak than those onto an angled impact surface. This is worth consideration for researchers trying to recreate the longer duration kinematics such as rotational velocity or brain strain dynamics.

Changing the impact surface angle resulted in minor changes to the rotational acceleration profiles. Because equivalent orientations were used on the two surfaces, differences were diminished to isolate the effect of changing the impact surface angle. Logically, larger differences would likely exist when considering impact orientations only achievable using an angled impact surface (such as the side rear boss orientation^53^). The largest differences were observed post-peak with impacts onto the flat surface creating lower magnitude secondary peaks in the forehead orientation, and larger secondary peaks in the rear orientation. The side orientation did not display any significant differences between flat and angled impacts across any kinematic profiles.

The rotational velocity profile was most heavily affected by the inclusion of a neck. Only minor changes in the rotational velocity manifested between the MEP pad and steel impact surface. Peaks occurred at similar times in the impact, and both followed the same shape with the main features temporally aligned. When the neck was included, rotational velocity peaks greatly increased in duration. This increase in duration was due to the increase in the allowable ROM offered by the neck. As this allowable ROM increased, larger rotational motions were achieved, resulting in longer duration rotational velocity peaks. A longer duration rotational velocity peak introduces a problem. Namely, the effect of a secondary impact on the rotational velocity profile. In this study, all secondary impacts occurred after the rotational velocity peak, however, this might not be the case for all impact test setups. Rotational velocities profiles were not greatly affected by the impact surface angle, however, in some cases (specifically the forehead), rotational velocity peaked much earlier on the angled impact surface compared to the flat surface.

### Peak Values

All peak kinematics were strongly correlated with the impact velocity and energy. PLA values differed vastly between drop test conditions with respect to both impact velocity and impact energy. Unsurprisingly, steel impacts resulted in the highest PLAs for a given impact energy/velocity. Similar to the linear acceleration profile differences, this was a direct result of the stiffness of the impact surface. The duration of steel impacts was shorter than those onto the MEP pad of the same impact energy, resulting in much higher PLAs. PLAs and PRAs followed much the same behavior, with steel impacts resulting in the highest PRAs and angled impacts resulting in the lowest.

Impacts with and without the neck onto a $0^\circ$ MEP pad displayed similar PLA and PRA values. Figures 5, 6 and 8–9 in Supplementary materials all show that including the neck did not affect the PLAs or PRAs. This implies that the peak values of the kinematics depend heavily on the impact surface stiffness and the impact surface angle, not the presence of a neck. Conversely, the neck had a significant effect on the shapes of the kinematic profiles. Interestingly, PRV values were mostly unaffected by the drop test condition and were strongly, linearly, correlated with impact velocity. The reasons for this are currently not known, but we hypothesise that head mass, and mass distribution, could have the greatest effect on PRVs.

As energy and velocity are proportional through the relation $E \propto v^2$ (or $E^\frac{1}{2} \propto v$), the regressions between impact velocity and energy are also related through the power parameter *b*. All regressions with respect to impact energy result in a power value that is half that of the regression with respect to impact velocity.

Perhaps of more relevance to recreating impacts in a laboratory setting are the ratios of peak values for each drop test condition. PLA and PRA were highly (nonlinearly) correlated, and this correlation was irrespective of the drop testing condition. This was not the case, however, for the PRV values. Although PRV was strongly correlated with PLA, there was a significant separation between the drop test setups used. Steel impacts resulted in the lowest PRV values for the PLAs produced, whereas angled MEP impacts resulted in the highest ratios of PRVs and PLAs. Similar results were seen when PRV and PRA were related, however, large ranges of PRAs seen across different impact orientations resulted in much less separation between the different impact setups. All regressions were forced to pass through the origin (0,0) as this was reasoned to make sense physically. An impact that has zero velocity or impact energy would be expected to produce zero acceleration and velocity (linear and rotational). For obvious reasons, this does not need validation.

### Comparison to Other Studies

Weight differences were excluded from this study as Gimble and Hoshizaki have already investigated this effect in 2008.^19^ These researchers found that, as head mass increased, the PLAs decreased for the same impact velocity. The effect of headform shape has also been studied in the past by Bland *et al.*^3^ These researchers found that in oblique impacts, HIII and NOCSAE headforms created significantly different PRAs and PRVs, with the NOCSAE headform producing 20–30% lower values, whilst the PLAs were not significantly influenced. The researchers also found large differences in peak values when carrying out angled impacts with and without a neck, with the no neck case resulting in peak values 17–35% higher than with a neck. This somewhat contrasts the results found in the present study, where the neck did not greatly influence the PLA and PRA values on a flat impact surface. It should be noted that the specific setup used in this study was different such that the headform was falling with the top of the head first in all impacts, thus complicating the comparison. Additionally, this study only tested impacts with an impact velocity of 6 m/s and did not quantify how these differences manifest across different impact energies.

Similar to the present study, Oeur and Hoshizaki also found a nonlinear increase in the PLAs and PRAs as the impact velocity increased.^46^ The authors also reported that impact surface compliance had the greatest effect on the peak accelerations, both linear and rotational. It should be noted that Oeur and Hoshizaki used a non-directional neck for their testing as opposed to the HIII neck used in the present study. Unfortunately, this study only compared the primary peaks of the acceleration curves and did not consider the rotational velocity. On top of this, only two impact velocities were used for the hardest impact surface, leading to a linear relationship between peak acceleration and impact velocity. Despite this, the results we present here strongly agree with those presented by Oeur and Hoshizaki.

### Limitations and Further Work

Several significant limitations exist within the present study. The most notable of which is the lack of investigation of the effects of the size and shape of the headform. We believe that increasing the size of the headform will greatly affect the rotational kinematics due to the different mass moments of inertia. Shape differences would also be expected to produce different rotational domain effects for the same reasons. Due to several restrictions, only drop testing could be carried out, and the effect of the drop carriage could not be quantified, however, we believe this will have a negligible effect on the initial peak and an increasing effect on any post-peak dynamics. Additionally, a consensus on why the PRV values were linearly correlated to the impact velocities could not be reached. Including impacts onto a flat and $45^\circ$ steel impact surface, with the neck, would strengthen the results, however the lightweight design of the testing components prohibited continued impacts onto steel. Future investigations using more robust testing equipment will aim to fill this gap. Lastly, we did not investigate how the drop test condition affects headgear performance. We believe that altering the drop test condition will significantly affect the reduction in peak kinematics associated with brain injury offered by the soft-shelled headgear used in rugby.

Outside the domain of rugby, a number of studies of hard-shelled headgear behavior and impact testing methods have been performed. A study of bicycle helmets by Abayazid *et al.*, and of ice hockey helmets by Levy *et al.* quantified the differences in potential injury reduction between helmet models using a finite element (FE) model of the brain. Thus could detail the effect of helmet use on simulated regional, and whole, brain strain.^2,36^ Recent advancements in machine learning models of brain strain have also allowed near instantaneous estimation of simulated brain strain without the need for a detailed FE model of the head.^18,60^ While these were not used in the present study, we aim to incorporate these into our future studies as indicative and comparative tools.

An extensive review of impact testing methods for headgear in sports conducted by Whyte *et al.* concluded that new headgear test methods primarily incorporate greater impact condition complexity while exploring more potential headgear assessment metrics than the respective standards.^57^ The authors continue by stating that most new impact test methods are unsuitable for a standards test in their current form due to their complexity. This, however, leads to several other related questions. Do we need to perfectly recreate impact conditions that exist on the field? What is the minimum comparative ‘realness’ needed to infer conclusions regarding the behavior of the headgear in a real-life impact situation? Finally, what metrics should be used, and in what ways, to quantify both beneficial and potentially negative effects of headgear use?

With the end goal of “improving player welfare”,^59^ headgear should be developed around a testing regime that reflects field conditions, with results of these tests quantified using metrics most strongly associated with brain injury. The first step is understanding how kinematics differ between different impact testing setups used by various research groups and testing standards. This study presents an analysis of four variations of the drop test method and quantifies the differences between them. We found that the impact surface stiffness greatly affected the PLAs and the duration of the peak. Inclusion of the neck significantly affected the rotational kinematics in both duration and kinematic profile. All peak kinematics were strongly correlated with impact velocity and energy. PLAs showed the highest variation between drop test conditions with steel impacts creating the highest linear accelerations, and 45° MEP impacts creating the lowest. Peak rotational accelerations showed less variance than linear accelerations between drop test conditions but held the same trends. Peak rotational velocities did not vary between drop test variations and were linearly correlated to impact velocity. The peak rotational accelerations were strongly correlated with the linear accelerations and did not vary significantly between impact conditions. The ratios of peak rotational velocity and peak linear velocity varied significantly between drop test conditions. Angled impacts produced the highest rotational velocities for a given linear acceleration, and steel impacts produced the lowest. The results of this study could influence the way headgear is tested, and how the headgear performance can be related to other studies and on-field performance. It can also provide a framework for recreating field measured impacts in the laboratory.

## Supplementary Information

Below is the link to the electronic supplementary material.

Supplementary file1 (PDF 174 kb)
