# Supplementary material for: Drop Test Kinematics Using Varied Impact Surfaces and Head/Neck Configurations for Rugby Headgear Testing
Source: Ann Biomed Eng. 2022 Aug 24;50(11):1633–47. doi: 10.1007/s10439-022-03045-5 (PMC9652288; doi:10.1007/s10439-022-03045-5)
Supplement: Supplementary file 1 — Supplementary file1 (PDF 174 kb) [file 10439_2022_3045_MOESM1_ESM.pdf]

2 Supplementary Figures

specific peak with impact velocity.png specific peak with impact velocity.png

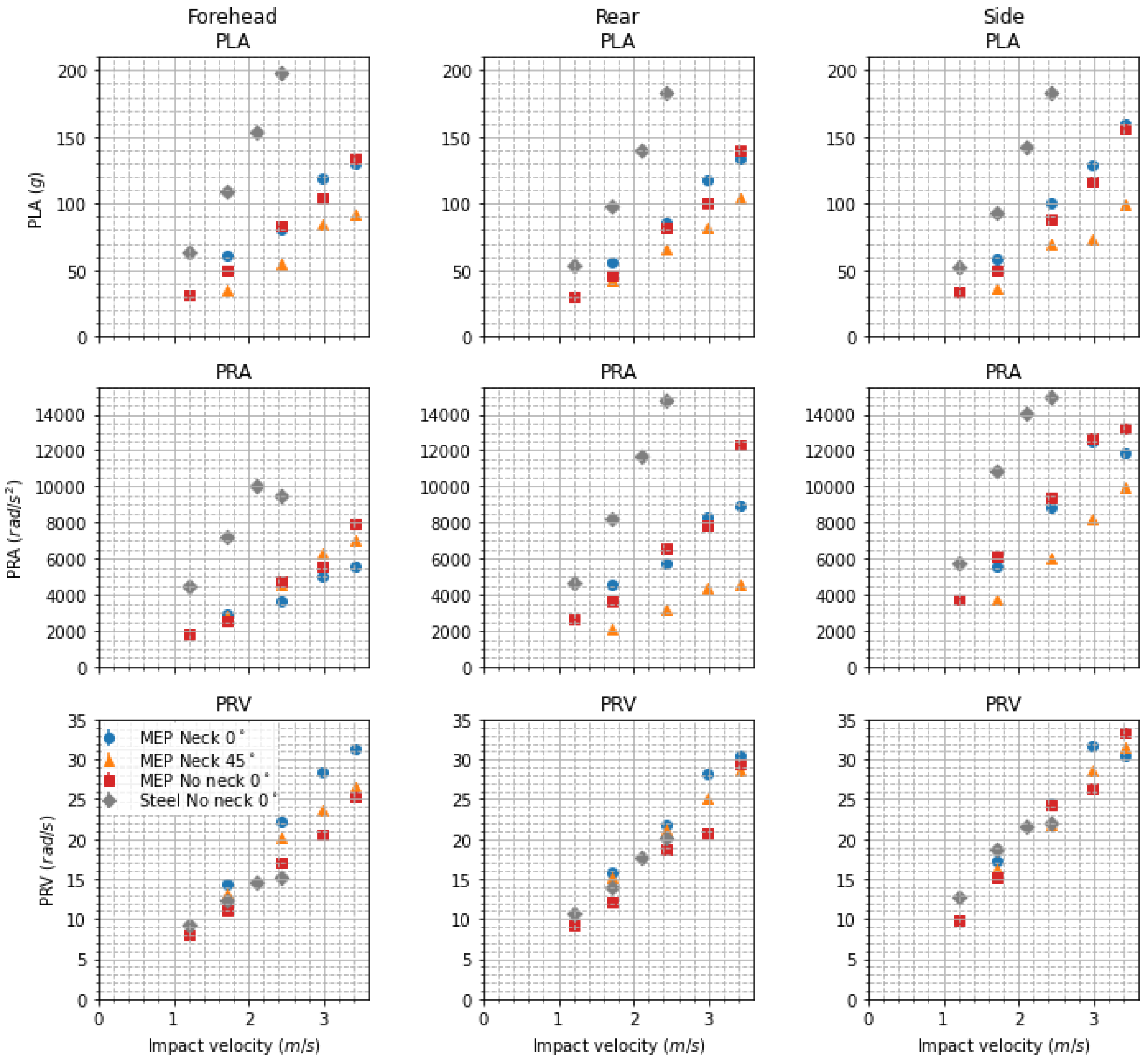

Figure 7: Orientation specific peak kinematic values with respect to impact velocity across each drop test condition.

specific peak with impact energy.png specific peak with impact energy.png

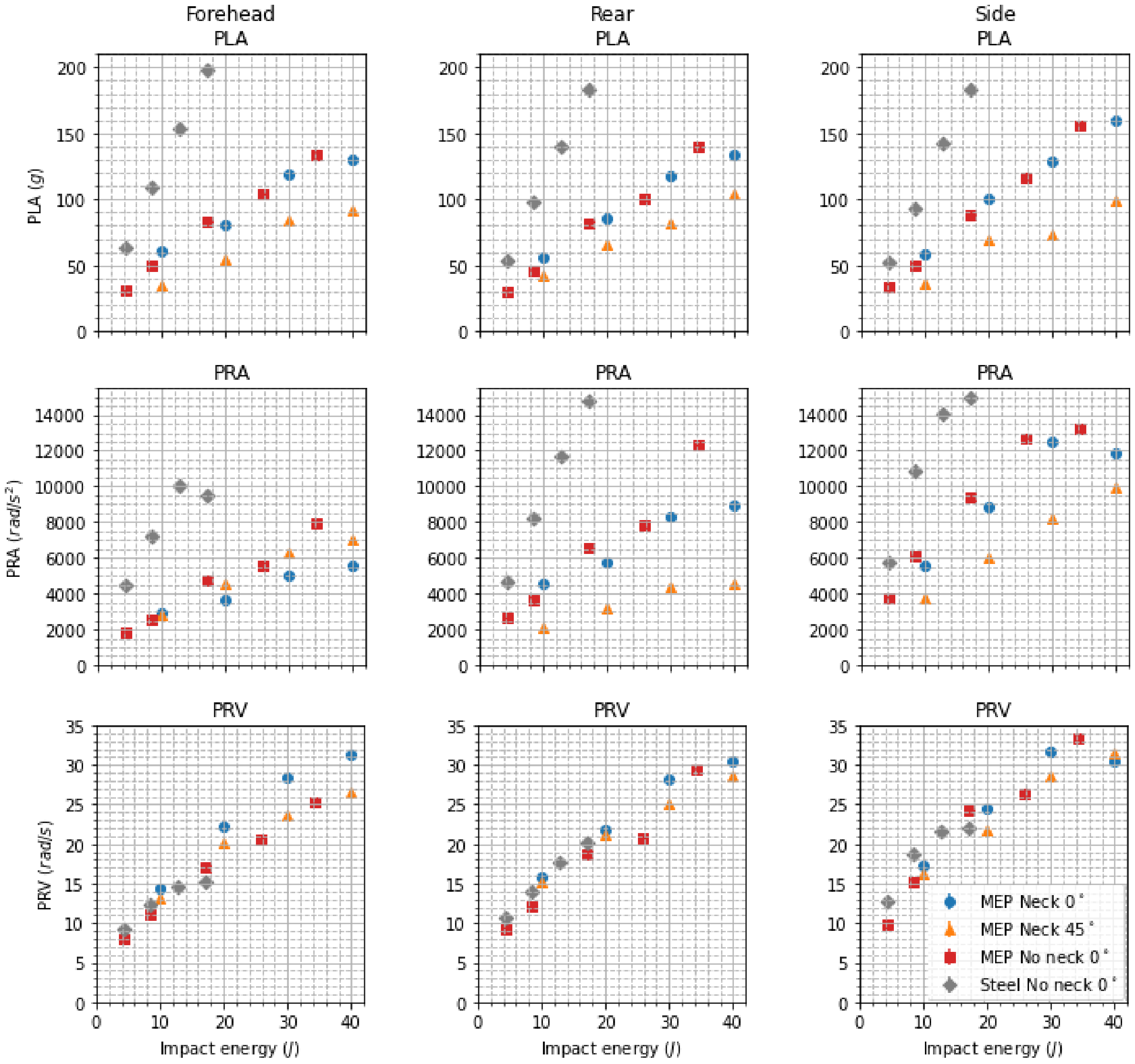

Figure 8: Orientation specific peak kinematic values with respect to impact energy across each drop test condition.

specific kinematics dependence.png specific kinematics dependence.png

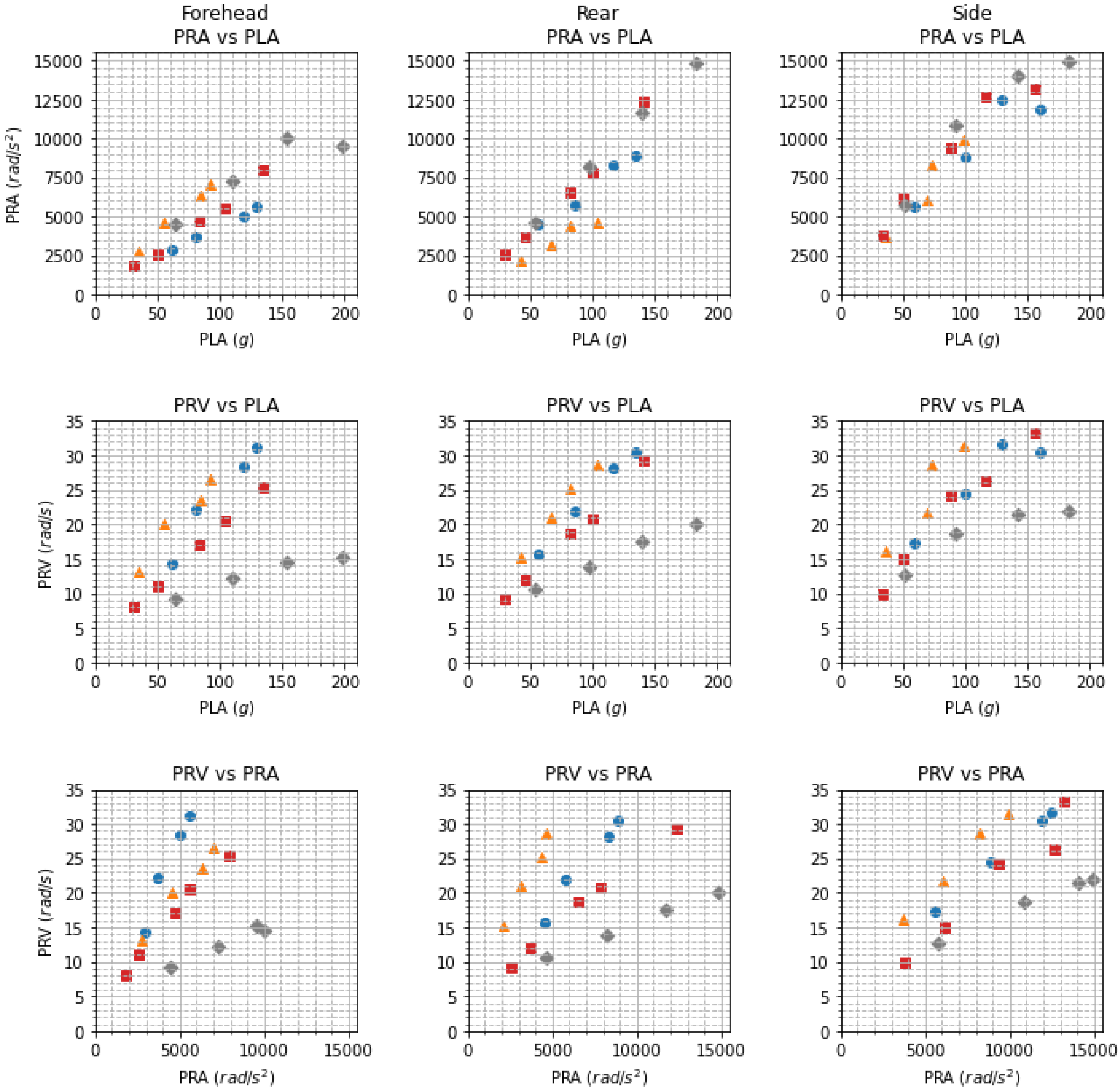

Figure 9: Figure shows how the peak kinematic variables relate to each other across all impact conditions for each orientation.
